# Functionally Graded Al_2_O_3_–CTZ Ceramics Fabricated by Spark Plasma Sintering

**DOI:** 10.3390/ma15051860

**Published:** 2022-03-02

**Authors:** Eszter Bódis, Miklós Jakab, Krisztián Bán, Zoltán Károly

**Affiliations:** 1 Institute of Materials and Environmental Chemistry, Research Centre for Natural Sciences, Magyar Tudósok krt. 2, H-1117 Budapest, Hungary; karoly.zoltan@ttk.hu; 2 Research Centre of Engineering Sciences, Department of Materials Sciences and Engineering, University of Pannonia, P.O. Box 158, H-8201 Veszprém, Hungary; jakab.miklos@mk.uni-pannon.hu; 3 Department of Automobiles Technologies, Faculty of Transportation Engineering and Vehicle Engineering, Budapest University of Technology and Economics, Műegyetem rkp. 3, H-1111 Budapest, Hungary; krisztian.ban@auto.bme.hu

**Keywords:** Al_2_O_3_–ZrO_2_ composite, functionally graded material, porosity

## Abstract

We studied the fabrication of functionally graded Al_2_O_3_–CeO_2_-stabilized-ZrO_2_ (CTZ) ceramics by spark plasma sintering. The ceramic composite exhibits a gradual change in terms of composition and porosity in the axial direction. The composition gradient was created by layering starting powders with different Al_2_O_3_ to CTZ ratios, whereas the porosity gradient was established with a large temperature difference, which was induced by an asymmetric graphite tool configuration during sintering. SEM investigations confirmed the development of a porosity gradient from the top toward the bottom side of the Al_2_O_3_–CTZ ceramic and the relative pore volume distributed in a wide range from 0.02 to 100 µm for the samples sintered in asymmetric configuration (ASY), while for the reference samples (STD), the size of pores was limited in the nanometer scale. The microhardness test exhibited a gradual change along the axis of the ASY samples, reaching 10 GPa difference between the two opposite sides of the Al_2_O_3_–CTZ ceramics without any sign of delamination or cracks between the layers. The flexural strength of the samples for both series showed an increasing tendency with higher sintering temperatures. However, the ASY samples achieved higher strength due to their lower total porosity and the newly formed elongated CeAl_11_O_18_ particles.

## 1. Introduction

Functionally graded materials (FGM) have gained increasing attention over recent decades due to the diverse fields of potential applications such as in engineering [1] and biomedicine [2,3]. By definition, FGMs [4] are characterized by a gradual transition of the composition and/or physical properties of the material along the bulk in one or more directions. It results in bulky materials having different behaviors in the various parts of the material tailored to the application. FGMs can be classified mainly into continuous and step-graded types depending on the interface conditions, but definite interfaces cannot be observed in either case. In this regard, the local concentration of stress, which may induce delamination within the material, can be avoided [5].

Plenty of examples can be found for gradient structures in nature including the human body. Because of this, intensive research is carried out to mimic such structures and develop functionally graded ceramics appropriate for various biomedical applications (e.g., bones and teeth). Currently, there are numerous potential materials for biomedical applications, including hydroxyapatite (HAp), bioglass [6,7], alumina, and zirconia [2]. However, all of them suffer from some shortcomings when used as a standalone monolithic material. Hap and bioglass have very poor mechanical properties, which limit their use in replacing bones. From that point of view, Al_2_O_3_ and ZrO_2_ provide potential benefits because of their biocompatible and much better mechanical properties than the above-mentioned materials, and they are already in use in various medical areas such as bone or dental implants [8,9]. Al_2_O_3_ is preferred due to its good biocompatible and hypoallergenic features as well as high hardness, however, it possesses low strength and fracture toughness. ZrO_2_ outperforms Al_2_O_3_ with respect to mechanical properties such as fracture toughness and strength, but it has low hardness and high density. However, Al_2_O_3_–ZrO_2_ composites provide a solution, as they combine the advantages of both ceramics.

The superior mechanical properties (high strength and fracture toughness) of stabilized zirconia stem from its unique phase transformation toughening mechanism [10]. Y_2_O_3_ is a widely applied stabilizer of the tetragonal ZrO_2_ (Y-TZP), but it is prone to time-dependent tetragonal (t)→ monoclinic (m) phase transformation, especially under aqueous conditions, which is also called low-temperature degradation (LTD). It may lead to difficulties during operation, e.g., on the surface of the material [11,12]. However, several studies reported that CeO_2_ stabilized tetragonal ZrO_2_ (CTZ) shows complete resistance to LTD under a humid atmosphere such as in an oral environment [13,14,15], and it also has significantly higher mechanical strength than Y-TZP [14,16]. Recently, an innovative bioceramic Al_2_O_3_ composite with CeO_2_ stabilized ZrO_2_ was reported as a degradation-free material [13,14,17]. Furthermore, laminated Al_2_O_3_–CTZ FGM composites can serve as biocompatible, as well as high-strength and high-toughness materials [8,14,18]. The surface containing 100% Al_2_O_3_ offers the biocompatible layer, while the ZrO_2_ content gradually increasing along the cross section provides the increased tensile strength and fracture toughness of the FGM.

Several methods for the fabrication of Al_2_O_3_–CTZ FGM have been reported in the literature including ceramics inkjet printing [19], tape casting, electrophoretic deposition [20], traditional [21], as well as a novel powder metallurgy technique, spark plasma sintering (SPS) [22]. Cai et al. [23] and Sun et al. [24] successfully produced layered Al_2_O_3_–CTZ FGM by traditional powder sintering and studied the optimal processing parameters to avoid cracks between the layers. Other authors [25] emphasized the study of creeping mechanisms in FGM between the layers. Salahi et al. [21] fabricated dual-, triple-, and quintuple-layered Al_2_O_3_–CTZ samples with different compositions and confirmed that the hardness and fracture toughness of the multi-layer Al_2_O_3_–ZrO_2_ structure was higher than those of the double-layer structure. Marshall et al. [26] fabricated laminar Al_2_O_3_–CTZ composites and showed a strong interaction between the layers. They proved it with R-curve behavior and a 3.5 times higher fracture toughness compared with the starting values of the composite. Anné et al. [27] found almost doubled strength for graded Al_2_O_3_–CZT compared with pure Al_2_O_3_. According to the above considerations, the Al_2_O_3_–CZT FGM composite seems to be very promising for biomedical applications.

The biocompatibility behavior of materials can be further improved by mimicking the porosity gradient structure of bone and teeth. The porous structure supports and controls the adhesion, growth, and proliferation of the cells in the ceramic, even by pores as small as a few nanometers (∼5 nm) in diameter [28]. Moreover, the adsorption, diffusion, and physical entrapment of proteins into the pores are expected to further enhance cell attachment and growth, and as a consequence, the biocompatibility of the material [29]. Various methods have been reported to develop a gradient pore structure in ceramics, such as the incorporation of pore-forming agents [30,31,32], gel- or freeze-casting [33], and electron deposition [20], among others. Recently, the SPS sintering method was successfully employed to develop porous materials [34].

SPS also offers an alternative route to create gradient microstructures by generating temperature gradients inside the sintered material during heating. The temperature gradient can be developed either in axial or radial direction, although the latter is negligible for non-conductive ceramics according to recent studies [35,36]. By contrast, either a specially designed graphite tool or an asymmetric graphite configuration can give rise to inhomogeneous current distribution axially, and as a consequence, the temperature gradient within the material [37,38]. The latter results in gradient properties through the cross section of the material in terms of either phase composition [39], chemical composition [40], or porosity distribution [41]. In our previous study [42], we also fabricated alumina with gradient porosity by creating a temperature gradient within the sample during SPS, using asymmetric graphite mold configuration.

In this study, our goal is to develop an Al_2_O_3_–CZT ceramic composite with gradually varying porosity and chemical composition. Composition is varied through four layers, while the porosity gradient is developed by in situ generated thermal gradient during SPS. This structure may provide mechanical properties comparable with that of human bones along with improved biocompatibility and bioactive features. Al_2_O_3_–CZT with gradient porosity can be a promising functional material in biomedical applications, since it combines good mechanical behavior with rapid osteointegration of the cells.

## 2. Experimental

### 2.1. Synthesis of Raw Nanoparticles

Ceria-stabilized (16 mol%) tetragonal ZrO_2_ nanoparticles were prepared by hydrothermal synthesis. The tetragonal phase stabilization and mechanical properties of ZrO_2_ ceramics are strongly affected by the CeO_2_ content [43,44] and it was found that CTZ ceramics possess enhanced strength and toughness with CeO_2_ content between 12–16 mol%. The precursors were zirconyle (IV) chloride hydrate (ZrOCl_2_·H_2_O) (Szkarabeusz Kft., Budapest, Hungary, 99.0%) and cerium (III) nitrate hexahydrate (Ce(NO_3_)_3_·6H_2_O) (Sigma- Aldrich Kft. Budapest, Hungary; 99.0%). ZrOCl_2_·H_2_O and Ce(NO_3_)_3_·6H_2_O were dissolved in 200 mL of distilled water for a CeO_2_ to ZrO_2_ molar ratio of 16:84. The Ce–Zr mixed salt solution was precipitated by adding 0.3 M ammonium hydroxide dropwise until the solution reached pH 10 and white gel was formed. The gel was filtered and washed with distilled water until chlorine ions (Cl^−^) could not be detected with a 0.03 M silver nitrate (AgNO_3_) solution. Finally, the gel was dried in an oven at 90 °C in air for 24 h, then calcined at 700 °C for 2 h. Nanosized Al_2_O_3_ was prepared by hydrothermal synthesis similar to CTZ; this route of synthesis is detailed in our previous study [42].

The calcined powders were investigated by X-ray diffraction (XRD) analysis, which confirmed that CTZ powder contains tetragonal crystals fitted by the JCPDF 38-1437 reference, whereas Al_2_O_3_ powder shows cubic crystal structured so-called γ–Al_2_O_3_ (JCPDF 10-0425). The average particle sizes were 90 nm and 70 nm for Al_2_O_3_ and CTZ powders, respectively, determined by scanning electron microscopy (SEM) analysis.

### 2.2. Preparation and Fabrication of 4-Layered Al_2_O_3_–CTZ FGMs

**Figure 1:**
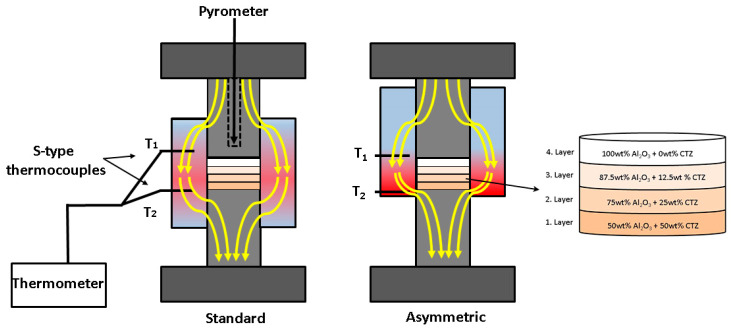
Illustration of temperature distribution and sample arrangement in standard and asymmetric graphite configurations in SPS.

To provide a gradual change in the composition of the ceramic, we prepared four powder mixtures with varying Al_2_O_3_ to CTZ ratios, as shown in Table 1. The composition of each layer was chosen based on the results of previous research [14,45]. The powder mixtures were homogenized in a Fritsch planetary ball mill with ethanol. Milling was performed in a Si_3_N_4_ tank with Si_3_N_4_ balls with a rotation rate of 500 rpm for 5 h. After milling, the powder was dried at 110 °C. The potential impurities stemming from the milling process were determined by EDXRF (energy dispersive X-ray fluorescent spectrometer) analysis method and silicon less than 0.5 wt% was also detected in the Al_2_O_3_–CTZ powder mixtures. The particular mixtures with different compositions were arranged in layers in the graphite sintering mold according to Figure 1. The amount of each mixture per layer was 2.8 g.

The layered powder mixture was consolidated with the use of SPS equipment (HD P5, manufactured by FCT System GmbH, Rauenstein, Germany) under vacuum at three different temperatures (1200 °C; 1250 °C; 1300 °C) controlled by the pyrometer of the SPS. The pyrometer measures the temperature of the graphite surface of the hole drilled in the upper graphite piston located a few mm above the sample surface as is shown in Figure 1.

The applied sintering conditions are shown in Table 2. The heating rate was 100 °C/min. The cooling rate was lowered to 30 °C/min to minimize thermo-mechanical stresses. The samples were fabricated in vacuum and a uniaxial pressure of 25 MPa was applied. The pulse cycle of the SPS machine was set to 12 ms on and 3 ms off; the characteristic time of each single pulse was 3.3 ms.

In addition to creating layers with different compositions in the sample, we also generated a gradient structure in terms of porosity, with temperature difference. We employed an asymmetric graphite configuration, where the powder was placed in an asymmetric position with respect to the graphite mold. This generated a temperature gradient within the sample. Sintering tests were also performed in standard graphite configurations (powders placed in the middle of the graphite mold) serving as reference materials. A schematic illustration of the sample positions is shown in Figure 1. Beyond the sintering conditions, the nominations of the samples are also given in Table 2, where the particular tests are referred by their letters indicating the graphite position (STD or ASY) followed by the temperature (e.g., ASY1200 stands for a sample sintered at 1200 °C in asymmetric graphite configuration).

In both cases, the local temperatures were monitored by two S-type thermocouples inserted into the inner side of the graphite mold. For the sake of reproducibility, each experiment was repeated three times. After the heat treatments, the average thickness of the samples was 6.3 ± 0.64 mm, whereas each layer was 1.58 ± 0.19 mm thick.

### 2.3. Analysis Methods

The samples were characterized for microstructure and mechanical properties. The total pore volume and pore size distribution of the sintered material were measured by mercury intrusion porosimetry (Poremaster 60 GT by Quantacrome Instruments). Determination of pore size by mercury intrusion porosimetry is based on the non-wetting behavior of liquid mercury. Mercury does not penetrate into pores by capillary force but only by applying external pressure. The required pressure is inversely proportional to the pore size. The relation between the pore size and the applied pressure, assuming cylindrical pores, is expressed by the Washburn equation:

(1) $$p=-\frac{2\gamma \cos\Theta }{r}$$

where *p* stands for the absolute applied pressure, r for the pore radius, γ for mercury surface tension (≈0.48 N/m), and *Θ* represents the contact angle (≈140°).

Due to the relatively long homogenization milling of the starting powder mixtures, the chemical composition of each batch was analyzed primarily for possible contamination. The analysis was performed by an energy dispersive X-ray fluorescence spectrometer (Thermo Scientific Niton Xl3t GOLDD+, Waltham, MA, USA) equipped with a Rh anode X-ray tube and energy-dispersive SDD detector, used at 15–50 kV and 30–120 µA, 120 s acquisition time.

We performed phase identification and determined structural characteristics of the as-prepared specimens by X-ray diffraction analysis (Philips PW 1830, Amsterdam, The Netherlands) using Cu Kα radiation, in the range of 10–70° 2θ in step-scanning mode with a step length of 0.04° for 1 s acquisition time per angle. The analysis was performed on the raw powders and on both surfaces of the sintered samples, as well. The fractured cross section of the samples was analyzed by SEM (FEI/ThermoFisher Apreo S LoVac, Waltham, MA, US) with the use of a secondary electron detector (SE) and backscattered electron detector (BSE), operated at 20 kV at various magnifications. Elemental analysis was also carried out on the samples by energy dispersion X-ray spectroscopy (EDX, Ametek Octane Elect Plus) during SEM measurements.

Among mechanical properties, hardness and flexural strength were determined. Hardness was measured by depth-sensing micro-indentation tests on the cut and polished surface of the samples, with a CSM2008 instrument (Peseux, Switzerland) equipped with a Vickers indenter tip. The load was applied for 15 s with a force of 500 mN. Hardness was calculated as the average of five indentation tests for all four sections of the sample. For each indentation, the load–penetration depth curve was automatically acquired and Vickers hardness was deduced from the unloading part of the load–depth curve. The flexural strength was measured with a three-point bending test. It was performed according to the AMMRC 85-21 standard (U.S. army standard test method for the flexural strength of high-performance ceramics at room temperature) with an Instron 5566 tester (Norwood, MA, USA) on three rectangular specimens.

## 3. Results and Discussion

### 3.1. Temperature Gradient during the SPS Process

**Figure 2:**
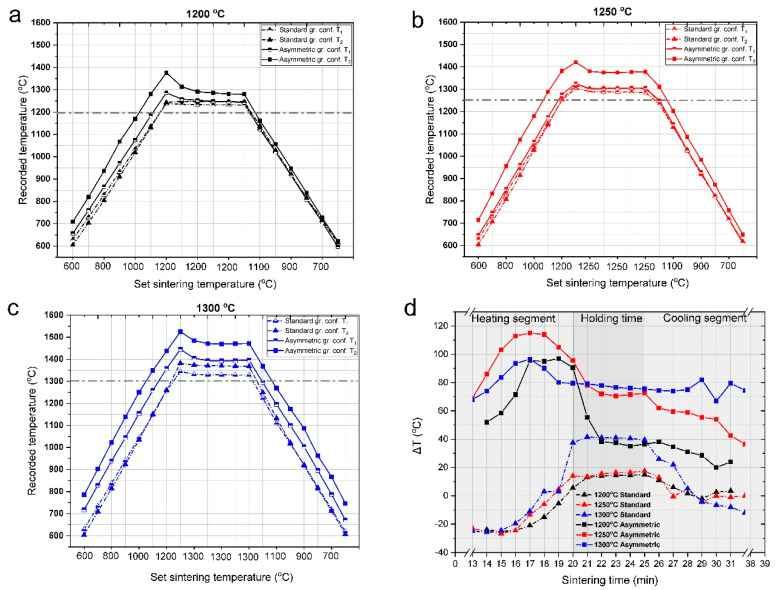
(**a**–**c**) Top (T_1_; full symbol) and bottom (T_2_; half symbol) temperatures of the samples as a function of pyrometer temperature. (**d**) represents ΔT as a function of sintering time for both configurations. The black, red, and blue curves represent sintering temperatures of 1200 °C, 1250 °C, and 1300 °C, respectively, where the solid line with triangle symbols displays the values in standard graphite configuration and the strain line with square symbols shows the asymmetric graphite configuration.

Gradient properties were created in the ceramic sample in two ways: (1) making up the bulk from layers of different composition, which leads to a chemical gradient, as well as (2) establishing a temperature gradient in the ceramic body during sintering, which results in a porosity gradient. The temperature gradient was generated by the asymmetric graphite configuration of the sample during the SPS sintering process, similarly to our previous study [42]. In Figure 2a–c, we illustrated the temperatures at the top (T_1_) and the bottom (T_2_) of the samples, recorded by the thermocouples as a function of pyrometer temperature for different sintering temperatures. For comparison, the temperatures of the reference sample, which was sintered in standard (non-asymmetric) graphite configuration, were also recorded and are shown in Figure 2. The temperature difference (ΔT) between T_1_ and T_2_ (T_2_ − T_1_ = ΔT) as a function of sintering time for both configurations (Figure 2d) is also illustrated.

The temperatures registered by the thermocouples were invariably above the set temperature measured by the pyrometer of SPS. The differences between the real and set values were higher for the asymmetric graphite configuration and increased with sintering temperature. (The horizontal dash lines indicate the set temperatures in Figure 2a–c). In the standard graphite configurations, the temperatures at the top of the sample (T_1_) were typically 40–70 °C over the set temperatures, while in the asymmetric configurations, the difference was twice as much. The temperature measured at the bottom of the sample (T_2_) was even higher, with a deviation of about 170 °C for sintering temperatures of 1200 °C and 1250 °C, and 200 °C for 1300 °C. These large temperature differences between the top and bottom of the dies can be explained by the inhomogeneous distribution of current density for the asymmetric graphite configuration during the SPS sintering process, as illustrated in Figure 1. Inhomogeneous distribution of the current density can also occur at the contacting points of the particles [46,47], leading to temperature differences inside the particles during sintering, as well.

Figure 2d shows ΔT generated between the top and bottom parts of the samples during the different sections of sintering. In the standard configuration, ΔT was not significant (the highest difference was 40 °C at a sintering temperature of 1300 °C) and alternates in the heating and sintering periods. However, in the asymmetric configuration, the temperature gradient was large and persisted throughout the sintering process. The largest ΔT was characteristic of the heating section reaching ca. 100 °C, probably due to the Joule heating effect, which is one of the typical heating mechanisms of SPS [48]. In the controlled cooling segments, the temperature difference decreased for both configurations, probably because there is less need for intensive heating, consequently, Joule-heating and current density decreased. However, for the samples made by asymmetric sintering, the detected temperature gradient remained until the end of the heat treatment process, whereas in the case of samples produced in the standard graphite configuration, ΔT approaches to 0 °C.

**Figure 3:**
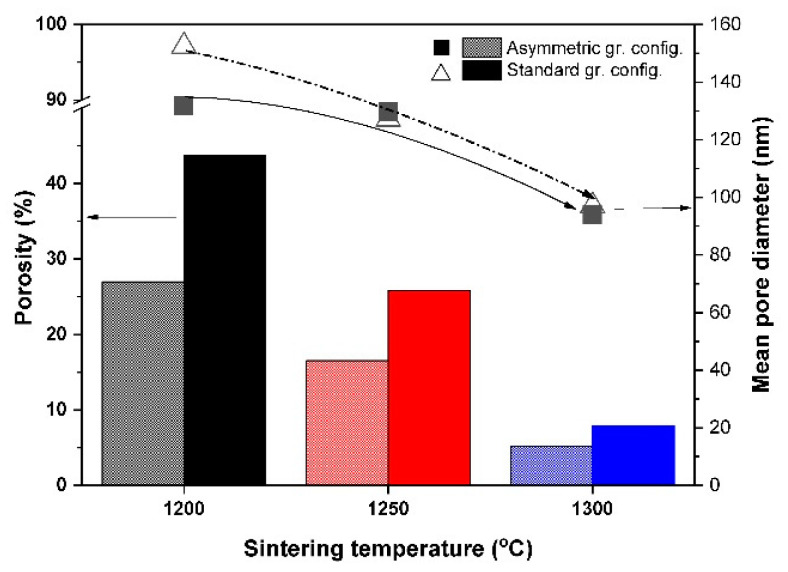
Total porosity and common pore diameter values of the samples fabricated at various temperatures in both graphite configurations. (Black, red and blue bars indicate the samples sintering at 1200 °C, 1250 °C and 1300 °C temperatures, respectively.)

The porosity and mean pore diameter values of the Al_2_O_3_–CTZ FGM sintered in various sintering conditions are shown in Figure 3. The porosity of the samples varied from 5% to 44%, with a rather wide range of pore sizes between 0.03 and 100 µm.

As shown in Figure 3, porosity decreases with increasing sintering temperatures for both configurations, but in the asymmetric graphite position, it reaches lower values than in the standard configuration, which could be expected considering the larger temperatures on both the top and bottom sides of the sample in the asymmetric position. For both series, porosity was greatly reduced to 5% and 8% for ASY1300 and STD1300 samples, respectively, even with a uniaxial pressure of only 25 MPa during the fabrication process. The mean pore diameter values of each sample followed a similar tendency—with increasing temperatures, mean pore size continuously decreased. The differences in the mean pore diameter between the two series were relatively small and became even smaller at higher sintering temperatures (Figure 3).

**Figure 4:**
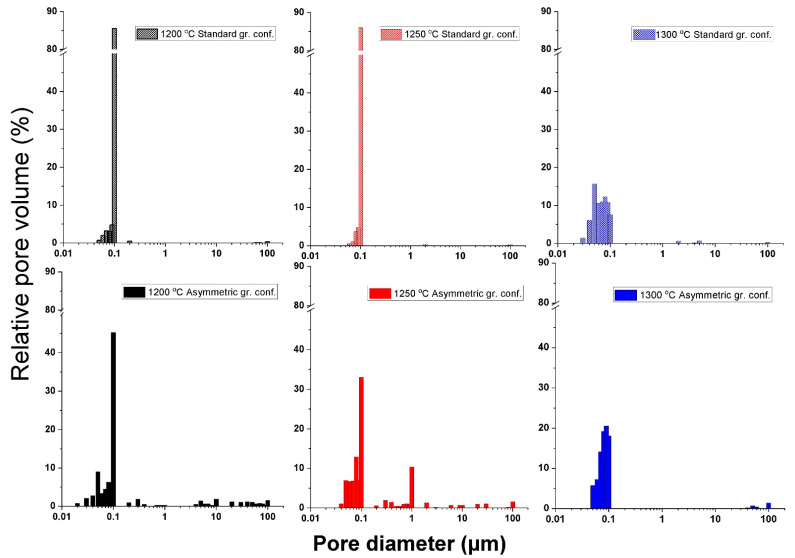
Total porosity distribution of the samples for both graphite configurations.

Figure 4, illustrates the pore size distribution of the sintered bulk ceramics. The samples showed remarkable differences in pore size distribution depending on SPS die configuration. The majority of the pores are around 0.1 µm but the ratio of this size range with respect to total pore volume is by far the highest in the STD series. At the highest sintering temperature (1300 °C), most of the smallest pores are eliminated due to the densification process, as indicated by the decrease in the relative pore volume from 85% to below 10% for 0.1 µm pores. As a result, pores of greatly different sizes are available in the sample.

In the ASY series, pores of about 0.1 µm in size also dominate regardless of sintering temperature, but their proportion is far smaller compared with the STD series and pores in the range of 0.03–100 µm can be also detected in a significant ratio even at the lowest sintering temperature (Figure 4). Based on several studies [49,50], the ideal pore sizes of biologically suitable materials vary in a wide range, from a few nanometers to tens of micrometers; therefore, we can assert that the obtained porosity structure of ASY samples may be suitable for biological applications. However, at the highest temperature (ASY1300), the larger pores are already negligible due to the much higher real temperatures characteristic of the asymmetric configuration. At these temperatures, the sintering process is accelerated; therefore, in the asymmetric configuration, the attained close to 1500 °C is too a high sintering temperature for a porosity-graded material.

### 3.2. Microstructure Analysis of the Al_2_O_3_–CTZ FGMs

**Figure 5:**
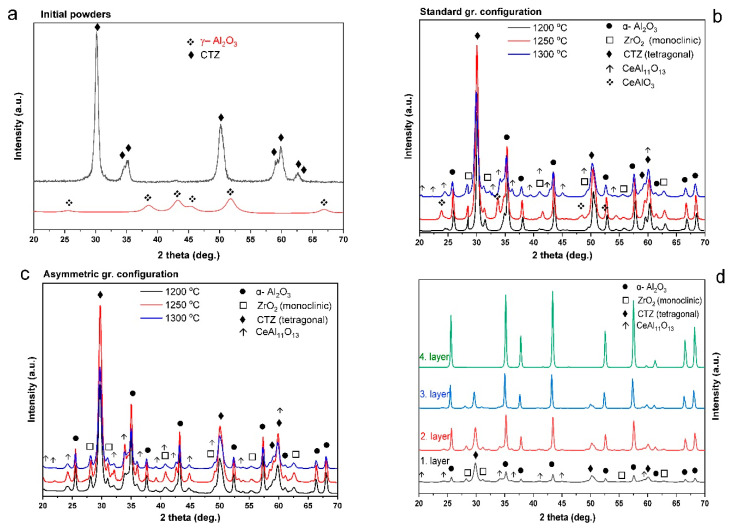
Phase composition of (**a**) initial powders; (**b**,**c**) STD and ASY series; and (**d**) each layer of sample ASY1200.

The phase composition of the green and sintered samples was analyzed by XRD. The analysis was carried out on the bottom side of the sintered samples, as well as on each layer. Figure 5a shows the XRD patterns of the initial Al_2_O_3_ and CTZ powders. The XRD analysis confirmed that γ-Al_2_O_3_ and tetragonal CTZ phases were obtained in good agreement with the JCPDS reference no. 00-010-0425 and no. 00-038-1437, respectively. Figure 5b,c shows the phase composition of the bottom side of the sample for both series, whereas Figure 5d illustrates the phase composition of each layer for sample ASY1200.

A comparison of the phase composition of the initial powders and the sintered samples reveals several changes. γ-Al_2_O_3_ transformed to the more stable α-Al_2_O_3_ phase (JCPDS no. 10-0173) in all samples, as the effect of the SPS heat treatment (Figure 5a–c). Furthermore, a small amount of monoclinic ZrO_2_ phase (JCPDS no. 37-1484) was also detected in the samples, regardless of the applied sintering conditions and configurations. However, tetragonal CTZ remained the major phase in the composition, predicting improved mechanical properties of the samples [14].

CeAl_11_O_18_ (JCPDS no. 48-0055) and CeAlO_3_ (JCPDS no. 81-1186) phases were also detected in minor amounts in certain samples. CeAlO_3_, supposing only as a transition phase in the formation of CeAl_11_O_18_, was present only in sample STD1250, while CeAl_11_O_18_ was present in STD1300 and in all samples of the ASY series. These phases were formed as a result of a solid-state reaction between the Ce_2_O_3_ and Al_2_O_3_ during the sintering process, based on previous observations [51]. We suppose that little CeO_2_ remained in the initial powder, which was reduced to Ce_2_O_3_ in the reductive atmosphere created by the high vacuum and the close contact of the powder mixture with the SPS graphite die. In sample STD1200, neither a CeAlO_3_ nor a CeAl_11_O_18_ phase was detected, but with increasing temperature, CeAlO_3_ was identified in small amounts at 1250 °C. The CeAl_11_O_18_ phase only appeared in the composition at high sintering temperatures (1300 °C), proving that it forms at high temperatures [52]. For the asymmetric graphite configuration, the CeAl_11_O_18_ phase appeared in all the samples, and its amount increased with sintering temperature shown by the increasing intensity of its diffraction peaks. Layer XRD analysis (Figure 5d) also showed a CeAl_11_O_18_ phase in the phase composition of each relevant layer.

**Figure 6:**
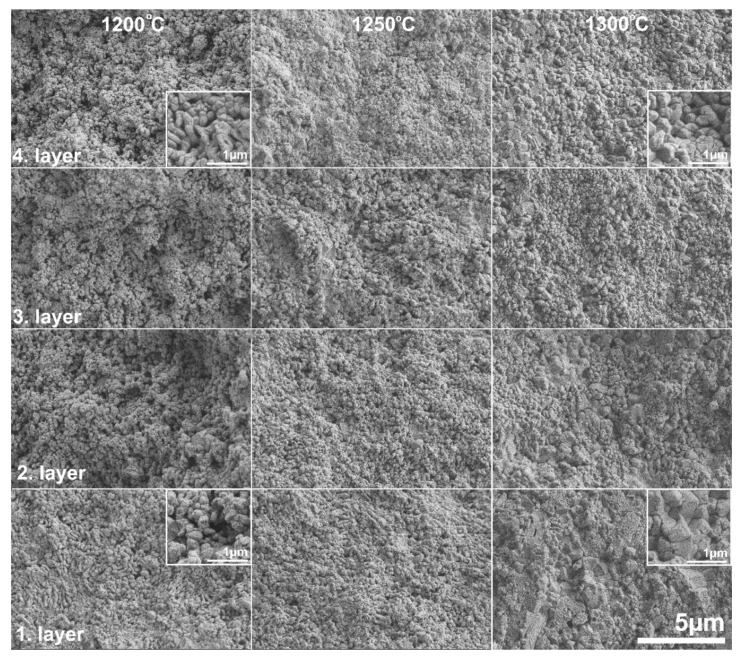
SEM micrograph of the fractured surface of the samples of STD series at various sintering temperatures.

**Figure 7:**
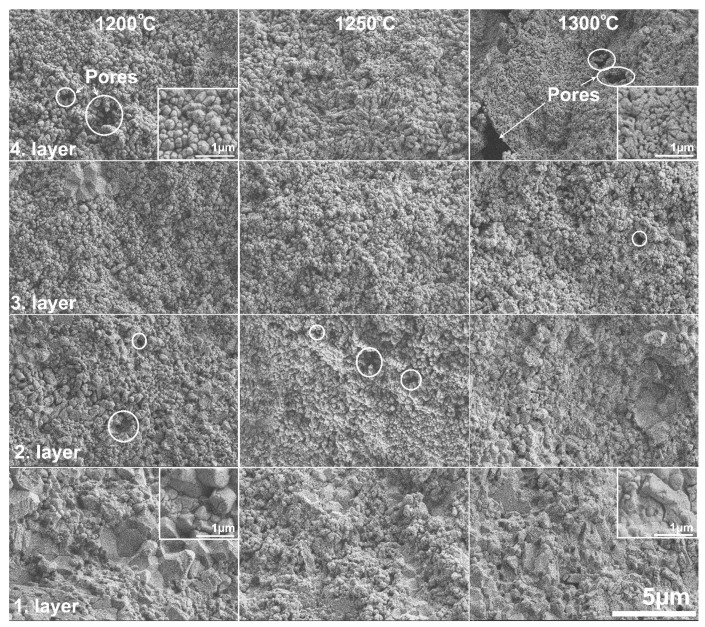
SEM micrograph of the fractured surface of the samples of the ASY series at various sintering temperatures. The white circles mark the pores in the structure.

The microstructure of the samples was investigated by SEM to determine the effects of (i) the standard and asymmetric graphite configurations and (ii) the applied sintering conditions on the microstructure of the samples. Figure 6 and Figure 7 show the cross section of the fractured surfaces of the samples sintered under different temperatures, revealing the structure of the different layers.

Figure 6 shows the cross section of the samples of the STD series. The microstructure unambiguously shows the porosity of the samples. The sample sintered at the lowest temperature (STD1200) shows a fine-grained but highly porous structure through all four layers without significant difference between the layers. However, notable changes can be observed with increasing sintering temperature. The porosity of the samples was significantly reduced along with grain coarsening. Grain size increased from 0.2 µm (STD1200) to 1–2 µm (STD1300). There are, however, no differences between the different layers.

In contrast, in samples sintered in the asymmetric configuration, a gradient structure can be revealed, as shown in Figure 7. This can be attributed to ΔT within the sample, which was as high as 100–120 °C. The microstructure changes gradually through the four layers, particularly in terms of porosity and grain size for all samples sintered at different temperatures. Even for sample ASY1200 sintered at the lowest temperature, significant differences are visible between the first and fourth layers. Whereas the first layer seemingly consists of 1–3 µm large grains with negligible nanometer-sized porosity, in the top part of the sample (fourth layer), 0.5–1 µm large pores are detectable among the 0.4–0.5 nm Al_2_O_3_ grains. (The pores were marked with white circles in the figures.) The differences are even more noticeable for the ASY1300 sample. The top (fourth) layer consists of 0.1–0.2 nm Al_2_O_3_ grains with large pores in the micrometer range, while the bottom (first) layer is almost pore-free containing 1 µm of large particles. SEM analysis also revealed that the pore structure comprises interconnected pores, particularly for the ASY1200 and ASY1250 samples.

We assume that the presence of silica impurity stems from homogenization of the starting mixtures by the Si_3_N_4_ balls, which may act as a sintering aid and promote the densification of the composites. At the grain boundary regions, binary or ternary eutectic phases with lower melting points could be formed in the Al_2_O_3_–SiO_2_–ZrO_2_ system, which contributed to the better sinterability of the composites, particularly for the ASY series having higher local temperatures at the bottom.

Consequently, the microstructural observations confirm that the asymmetric configuration contributed to a gradually increasing density towards the bottom part of the samples, resulting in reduced porosity in that region, while the top of the samples preserved their porous microstructure.

Additionally, several studies [35,36] point out the possible formation of a temperature gradient in the radial direction of the samples, but its magnitude strongly depends on the conductivity of the material. For non-conductive materials, only a temperature difference of 10–25 °C was observed. Our SEM studies found no evidence of a radial temperature gradient in the microstructure of the samples.

**Figure 8:**
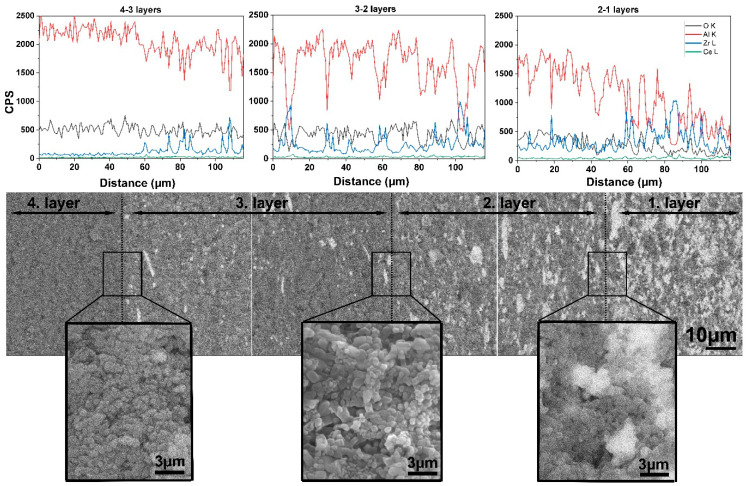
Cross section of SEM-BSE images with EDX analysis of Al_2_O_3_–CTZ FGM for sample ASY1200. Grey areas represent Al_2_O_3_ and bright areas represent Ce-ZrO_2_.

Elemental analysis was also performed through the cross-section of the samples by EDX. Figure 8 presents the distribution of Al, Zr, Ce, and O along the cross section of the ASY1250 sample, and its fracture surfaces shown by BSE-SEM. The Zr and Ce content gradually increased from the top (fourth layer) to the bottom (first layer) of the sample. At the same time, an even more pronounced decrease occurred in the Al content. The BSE images also reflect the gradual increase in the CTZ phase (bright parts) along the sample. High magnification images taken of the regions at the supposed boundary of the different layers are also shown in Figure 8. However, there are neither sudden changes nor definite interfaces between them. In other words, we cannot speak about different layers in the sintered samples but only one material with a gradually changing microstructure.

**Figure 9:**
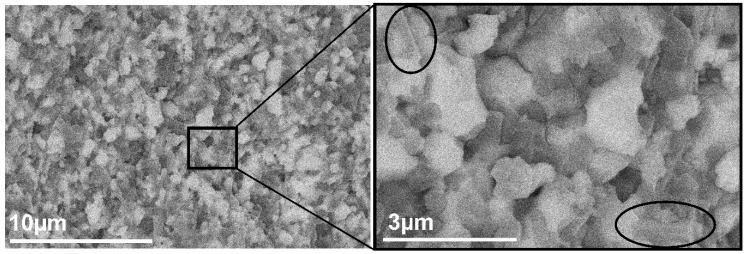
SEM-BSE image of Al_2_O_3_–CTZ FGM for first layer of sample ASY1300.

High magnification microstructure analysis revealed elongated particles in the structure. (They are shown with black circles in Figure 9). It is assumed that those particles belong to the CeAl_11_O_18_, since Akin et al. [51] and Kern at al [40] reported similar observations; furthermore, it also had a beneficial effect on the mechanical properties of the studied ceramics.

### 3.3. Mechanical Properties of Al_2_O_3_–CTZ FGMs

**Figure 10:**
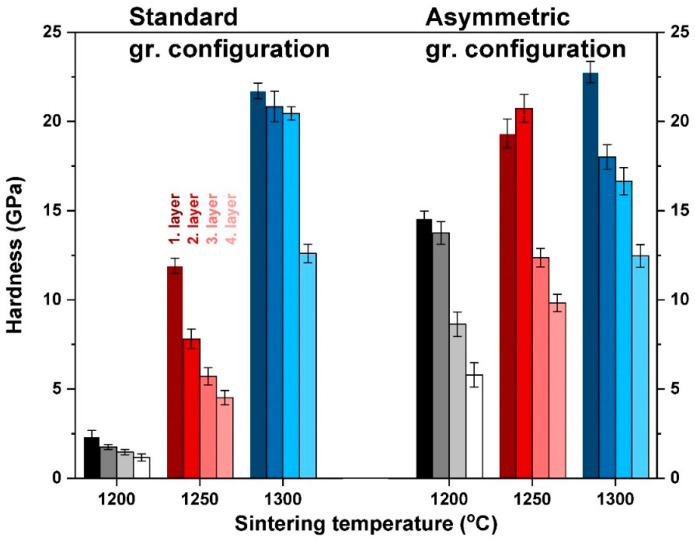
HV values of the samples for both configurations. (Black, red and blue bars indicate the samples sintered at 1200 °C, 1250 °C and 1300 °C temperatures, respectively.)

The mechanical properties of the composites were determined with microhardness and by three-point bending tests. Vickers indentation tests were carried out on all four layers of the samples to investigate the differences between the top and bottom sides of the samples accurately. In Figure 10, we compared the mean hardness values of both the STD and ASY series. Vickers hardness (HV) reflects gradual changes in the microstructure of all the samples.

As can be seen, increasing hardness with respect to increasing sintering temperature is characteristic of both series, which reflects the change in the porosity of the samples. Lower porosity results in greater hardness—this correlation between hardness and porosity is well known [53,54]. The hardness of the constituent layers with different compositions show a gradual trend at each sintering temperature. Interestingly, the greatest hardness is exhibited by the 50–50 wt% mixed layer, which contains the least Al_2_O_3_, even though, theoretically, Al_2_O_3_ is harder than CZT [42]. This phenomenon suggests that (i) hardness is basically determined by the microstructure and not only by the composition, and (ii) the porosity of the fourth layers is significantly higher than that of first layers. This may seem reasonable in asymmetric configurations, considering the higher temperature at the bottom of the sample, as Figure 1 illustrates. However, for standard configurations, such temperature deviation between the upper and bottom parts of the samples does not occur. In these cases, the higher porosity (and lower hardness) of the alumina-rich layers can be explained by the fact that the complete sintering of pure alumina requires a higher temperature than zirconia–alumina mixed phases. This way, the composition of the layers also affects ultimate porosity, and in turn, the hardness of the sample. In the asymmetric configuration, this combined effect of composition and temperature gradient can result in evenly distributed gradual changes in the hardness of the ASY series. The hardness of each layer is considerably greater compared with the STD series both at 1200 °C and 1250 °C. It can be explained by the differences in the higher temperatures during sintering. At a sintering temperature of 1300 °C, the two configurations also differed in the achieved peak temperature, and the higher temperature may result in enhanced hardness for the ASY series, too. However, it was not the case. Only minor differences were observed between the hardness values of the particular layers of the two series. It is not surprising, however, if we consider that the overall porosity of both samples was almost identical. The higher temperature of the ASY series was still not enough for the pure alumina layer to reach full densification. However, creating fully densified FGM was not our goal.

The evenly graded microstructure that can be attained at 1250 °C or 1300 °C by asymmetric sintering provides a more homogeneous distribution of HV within the sample. The formation of internal stress can be avoided between the consecutive layers, and this increases the load capacity of the samples. This is also confirmed by the microstructure, which has a smooth transition of composition and porosity without any sign of delamination at the imaginary interfaces of the layers (Figure 8).

**Figure 11:**
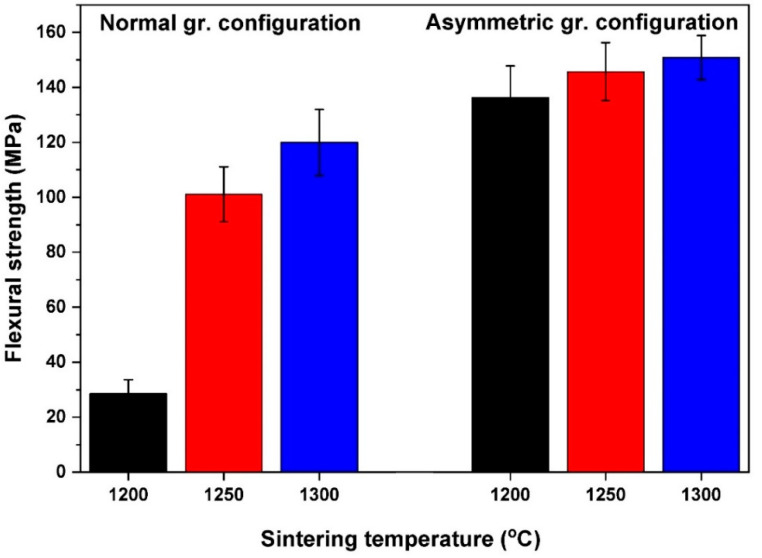
Flexural strength of the samples for both configurations. (Black, red and blue bars indicate the samples made at 1200 °C, 1250 °C and 1300 °C sintering temperatures, respectively.)

The flexural strength of FGM bodies was determined with three-point bending tests and the achieved values show a trend similar to HV and porosity. With increasing sintering temperature, flexural strength increases, too. Moreover, strength is further improved in asymmetric graphite configuration. The average strength values attained at different temperatures are illustrated in Figure 11 for both series.

The STD1200 sample has significantly lower flexural strength than all the other samples. The flexural strength of the ASY1200 sample is almost five times higher. The main reason for this huge difference is presumably the high porosity of the STD1200 sample. The strong correlation between strength and porosity is also known [43], but the almost identical total porosity does not mean equal strength. Although STD1250 and ASY1200 have nearly the same porosity as STD1300 and ASY1300, they significantly differ in strength, which was higher for the ASY series. This can be explained with (i) the different porosity distribution and (ii) the presence of CeAl_11_O_18_ phase. These particles have an elongated shape, as it can be observed in Figure 9, so they can provide a reinforcing effect, similarly to whiskers. The flexural strength of the FGM samples is less compared with the values of completely dense alumina, zirconia, or ZTA composites. However, similar values were reported by Kamyshnaya et al. [33] and Mangalaraja [55] for the ceramics they prepared with a composition and porosity identical to our samples. Moreover, the mechanical properties of the fabricated FGM composites also match those of natural bones [56,57], making the composite better suited for bone replacement applications.

## 4. Conclusions

We prepared functionally graded Al_2_O_3_–CTZ ceramics fabricated using spark plasma sintering. Owing to the thermal gradient generated during SPS consolidation, the obtained material exhibited a gradually changing composition and porosity in the axial direction. The following conclusions can be drawn:

- In the asymmetric graphite configuration, there is a significant temperature difference between the top and bottom part of the samples. The difference increases with increasing sintering temperatures and reaches as much as 120 °C at a sintering temperature of 1300 °C. At the same time, the temperature at the top side was also invariably higher than the set temperature.
- Mercury porosimetry and SEM analysis showed the formation of a gradient pore structure for the samples heat treated in an asymmetric configuration. Total porosity decreased with higher sintering temperatures, regardless of the type of graphite configuration. However, it was still lower in the asymmetric graphite configuration. At the same time, the relative pore volume was distributed in a wide range, and pores of up to 100 µm were detected in a significant ratio, as well.
- SEM analysis also showed that porosity and pore size distribution is anisotropic—densification increased toward the bottom part of the samples compared with the reference material. Microstructural analysis at higher magnification confirmed a smooth transition of the material in terms of composition and porosity without any sign of delamination or cracks at the imaginary interfaces of the layers. XRD and SEM analysis revealed that the newly formed CeAl_11_O_18_ phase in structure, which has an elongated appearance, presumably affected the mechanical properties of the FGM.
- Microhardness and flexural test results also indicate the changes in the composition and porosity of the Al_2_O_3_–CTZ material. Hardness increased gradually along the axis, particularly for the asymmetric configuration samples. A difference of nearly 10 GP in hardness was achieved between the top and bottom part of the sample heat treated at 1300 °C. For these samples, hardness distribution was more homogenous within the sample, which prevents the formation of internal stresses inside the materials, therefore providing better load capacity.
- The flexural strength of the samples for both series shows an increasing tendency with higher sintering temperatures. However, the samples sintered in asymmetric configuration achieved significantly higher strength due to the lower total porosity of the sample on one hand, and the newly formed elongated CeAl_11_O_18_ particles on the other. The FGM materials produced possess better mechanical properties than natural bones.

In summary, the present results suggest that the functionally graded composite material of Al_2_O_3_–CTZ is very promising for biological applications because the high-porosity portion of the FGM allows fast bone ingrowth, while the low-porosity part can withstand high mechanical stresses.

## Figures and Tables

**Table 1:** Phase composition of each layer.

Layer	Composition (wt%)	
	Al_2_O_3_	CTZ
4.	100	0
3.	87.5	12.5
2.	75	25
1.	50	50

**Table 2:** Applied sintering conditions and the nomination of the samples.

Sintering Conditions					
Temperature (°C)	Pressure (MPa)	Graphite Configurations			
		Standard (STD)	Sample name	Asymmetric (ASY)	Sample name
1200	25		STD1200		ASY1200
1250			STD1250		ASY1250
1300			STD1300		ASY1300

## Data Availability

The data that support the findings of this study are available from the corresponding author.
